# A Handbook of Hospital Practice

**Published:** 1861-01

**Authors:** 


*A Handbook of Hospital Practice, or an Introduction to the Practical Study of Medicine at the Bedside.* By ROBERT D. LYONS, late Pathologist-in-Chief to the British Army in the Crimea, &c., &c. S. S. & W. Wood, 389 Broadway, N. Y.—*Publishers.*

This handsome little volume, of 175 pages, has just been received from the publishers, and we are sorry to have no time for an extended notice in this number of the Journal. In the work we find full directions for "Preliminary Examinations," general examinations, and special examinations of the different organs, in order to determine the character of diseases. Fifty-one pages are devoted to the manner of investigation in pathology by post mortem examinations.—In the Appendix are found ample directions for writing prescriptions; after which, follows a glossary, and the work concludes with forms for reporting cases. It is altogether a useful little work, and we think no subjects can be more profitably investigated, by the physician and advanced student of medicine, than those treated of by the author.

